# Oral Prescription Containing Ceramide, Proanthocyanidins, Quercetin, and Citrus Flavonoids Improves Sensitive Skin in Mice by Modulating Inflammation, Skin Barrier Function, and Gut Microbiota

**DOI:** 10.1111/jocd.70468

**Published:** 2025-12-15

**Authors:** Miaohong Yang, Chaoyue Wang, Yaqi Yang, Ping Han, Ling Jiang, Shanpeng Chu, Guangling Yin, Lingna Xie, Jieqiong Chen, Xiaohui Yang, Zhiyun Du

**Affiliations:** ^1^ School of Biomedical and Pharmaceutical Sciences Guangdong University of Technology Guangzhou Guangdong P.R. China; ^2^ BYHEALTH Institute of Nutrition & Health Guangzhou Guangdong P.R. China

**Keywords:** ceramide, citrus flavone, gut microbiota, procyanidins, quercetin, sensitive skin, skin barrier functionatopic dermatitis

## Abstract

**Background:**

Sensitive skin is characterized by impaired skin barrier function, inflammation and dryness, and the effects of existing treatments are limited. This study explored an oral prescription containing ceramides, proanthocyanidins, quercetin, and citrus flavonoids, which inhibit inflammation, enhance barrier function, and regulate the gut‐skin axis through a multi‐target mechanism to improve skin sensitivity.

**Methods:**

The therapeutic effects of the oral prescription were evaluated using the ‐induced AD model and the AEW‐induced dry skin model. Key endpoints included skin lesion severity, TEWL, skin thickness, mast cell infiltration, inflammatory cytokine levels, and skin barrier protein expression. Microbial changes in gut flora were also assessed to evaluate the therapeutic effects of the gut‐skin axis on sensitive skin.

**Results:**

In the AD model, oral high‐dose prescription (PG‐H) significantly improved skin lesions, reduced TEWL, epidermal thickness, and ear swelling, decreased mast cell infiltration and inflammatory factors (IL‐4/6/31, ET‐1), and enhanced barrier hydration. In the AEW model, PG‐H reduced TEWL, alleviated dryness and scaling, and upregulated the expressions of filaggrin, AQP3, and loricrin, indicating a restoration of skin barrier function and relief from skin dryness. Furthermore, PG‐H regulates the intestinal flora (with a reduction in Firmicutes/Bacteroides) and suggests potential therapeutic benefits for skin health.

**Conclusions:**

The oral formulation containing ceramides, proanthocyanidins, quercetin, and citrus flavonoids improves skin barrier function, reduces inflammation, restores skin hydration, and modulates the skin‐gut axis through multi‐pathway and multi‐target mechanisms, effectively alleviating symptoms of atopic dermatitis and dry skin, thus highlighting its potential as a novel dietary supplement for treating sensitive skin and related conditions.

**Abbreviations**
AEW: acetone‐ether‐water
AQP3: aquaporin 3
DNFB: 1‐chloro‐2，4‐dini‐trochlorobenzene
ELISA: enzyme‐linked immunosorbent assay
ET‐1: endothelin‐1
FLG: filaggrin
HE: hematoxylin–eosin staining
IFN‐γ: interferon‐γ
IL‐13: interleukin‐13
IL‐4: interleukin‐4
IL‐6: interleukin‐6
LOR: loricrin
TEWL: trans epidermal water loss
TRPV1: transient receptor potential vanilloid 1
VEGFA: vascular endothelial growth factor A

## 1 Introduction

The pathogenesis of sensitive skin involves the interaction of various cytokines and receptor pathways [1, 2]. Pro‐inflammatory factors such as IL‐4, IL‐6, IL‐31, and IFN‐γ exacerbate inflammatory responses and barrier damage, while the activation of IL‐31 and TRPV1 receptors leads to neurogenic itching and burning sensations. ET‐1 aggravates skin flushing and sensitivity through vasoconstriction and inflammation [3, 4, 5, 6, 7]. Additionally, gut microbiota dysbiosis affects immune balance via the “gut‐skin axis” potentially elevating IgE levels and pro‐inflammatory factor expression, further intensifying skin sensitivity [8, 9, 10]. These factors collectively contribute to the inflammation, barrier impairment, and neurovascular abnormalities observed in sensitive skin. However, current treatments for sensitive skin are relatively limited and fail to comprehensively address the multifaceted aspects of inflammation, barrier dysfunction, neural responses, and gut microbiota. Additionally, some medications, such as corticosteroids, may cause side effects or dependency [11, 12, 13]. Given the current challenges in effectively treating and curing sensitive skin, the development of novel dietary supplements for the treatment of sensitive skin is of significant importance. Several plant extracts, including proanthocyanidins, quercetin, and citrus flavonoids, have shown potent anti‐inflammatory, antimicrobial, and skin barrier‐protective properties, making them promising candidates for the treatment of sensitive skin [14, 15]. Proanthocyanidins, derived from sources such as grape seeds and pine bark, are renowned for their potent antioxidant and anti‐inflammatory properties, which help reduce skin oxidative stress and inflammation through mechanisms such as scavenging free radicals and inhibiting the NF‐κB signaling pathway [16, 17]. Quercetin, a flavonoid found in fruits such as apples and onions, has demonstrated significant potential in reducing the production of pro‐inflammatory cytokines (TNF‐α, IL‐6), inhibiting the release of inflammatory mediators, and alleviating atopic dermatitis (AD) and skin irritation [18, 19]. Citrus flavonoids, such as those found in oranges and lemons, have been shown to promote skin hydration and restore barrier function by enhancing collagen synthesis and promoting keratinocyte differentiation [20, 21]. Ceramides are lipids naturally present in the skin that replenish stratum corneum lipids and enhance intercellular adhesion among keratinocytes, playing a crucial role in maintaining skin barrier function and reducing TEWL [22, 23]. Combining these ingredients provides a multi‐targeted therapeutic approach, effectively alleviating inflammation, improving barrier dysfunction, and mitigating oxidative stress, all critical factors contributing to sensitive skin [24]. Most existing studies have focused solely on a single aspect, failing to address the repair and treatment of sensitive skin from a comprehensive perspective. Currently, research on improving skin sensitivity through plant‐based combinations that exert multi‐pathway and multi‐targeted mechanisms to suppress inflammation, enhance skin barrier function, and modulate the gut‐skin axis remains relatively limited.

This study employs DNFB‐induced atopic dermatitis and AEW‐induced dry skin models to investigate the multi‐pathway and multi‐targeted effects of a plant‐based combination (comprising ceramides, proanthocyanidins, quercetin, and citrus flavonoids) on skin sensitivity, focusing on its ability to suppress inflammation, enhance skin barrier function, and modulate the gut‐skin axis, thereby elucidating the underlying regulatory mechanisms of skin sensitivity.

## 2 Materials and Methods

### 2.1 Reagents and Chemicals

DNFB, acetone, ether, olive oil, dexamethasone, physiological saline were purchased from Aladdin (Shanghai, China). Konjac flour (3% ceramide), grape seed extract (95% proanthocyanidins), *Sophora japonica* extract (95% quercetin), and citrus flavone (45%) were purchased from BY HEALTH Institute of Nutrition and Health. IL‐4, IL‐6, IL‐31, IFN‐γ, ET‐1, VEGFA, and TRPV1 ELISA kits were bought from JiangLai Biotechnology (Shanghai, China). The BCA protein assay kit was obtained from Beyotime Biotechnology (Shanghai, China).

### 2.2 Animal

SPF BALB/c mice aged 6–8 weeks, 18–22 g, male, six in each group, provided by Guangdong Animal Center. All mice were kept in the animal room of the Experimental Animal Center of the School of Biomedicine, Guangdong University of Technology. The indoor temperature of the animal room was maintained at 20°C–25°C and the humidity was 45%–65%. The mice were fed freely for 1–2 weeks with a 12‐h light/dark cycle lighting system.

### 2.3 Atopic Dermatitis Model Induced by DNFB

These mice were randomly divided into six experimental groups: normal control group (NC), DNFB model group (DNFB), dexamethasone treatment group (DEX), low‐concentration prescription group (PG‐L), medium‐concentration prescription group (PG‐M), and high‐concentration prescription group (PG‐H). The composition and proportion of the prescription are shown in Table 1. The back hair of BALB/c mice was removed before the experiment. Two days later, the model was made on Day 1, with 0.15% DNFB 100 μL applied to the back and 25 μL applied to the right ear. The blank group was applied with acetone olive oil (v:v = 3:1) and was applied again on Day 4, twice in total. Day 7 began to stimulate, and 0.2% DNFB 100 μL was applied to the back, and Day 10 and Day 13 were applied again for three times, making an atopic dermatitis model [25]. The drug was administered daily after the experiment began. Mice in the DNFB model group and the normal group were given 200 μL normal saline intragastrically, while mice in the experimental group were given corresponding concentration of prescription once a day for 14 days.

**TABLE 1:** The composition and content of different prescription.

Grouping	Composition	Concentration (mg/mL)
PG‐L	Konjac flour (3% ceramide)	0.91
	Grape seed extract (95% proanthocyanidins)	3.03
	*Sophora japonica* extract (95% quercetin)	6.07
	Citrus flavone (45%)	7.58
PG‐M	Konjac flour (3% ceramide)	1.82
	Grape seed extract (95% proanthocyanidins)	6.07
	*Sophora japonica* extract (95% quercetin)	12.13
	Citrus flavone (45%)	15.17
PG‐H	Konjac flour (3% ceramide)	2.73
	Grape seed extract (95% proanthocyanidins)	9.10
	*Sophora japonica* extract (95% quercetin)	18.20
	Citrus flavone (45%)	22.75
DNFB	Dexamethasone	0.30

### 2.4 Model of Skin Dryness Induced by AEW

These mice were randomly divided into six experimental groups: normal control group (NC), AEW model group (AEW), dexamethasone treatment group (DEX), low‐concentration prescription group (PG‐L), medium‐concentration prescription group (PG‐M), and high‐concentration prescription group (PG‐H). Hair was removed from the back of the BALB/c mice. Two days later, the model was established. The mice's back were soaked with cotton balls of acetone/ether (1: 1) mixture for 30 s, then soaked with distilled water cotton balls at the same position for 30 s, while the blank group was soaked with distilled water cotton balls for 60 s, twice a day (9:00 and 17:00), and were smeared and soaked continuously for 7 days. A mouse model of dryness, itching and allergy was made [26]. Give the drug a week in advance. Mice in AEW model group and normal group were given 200 μL normal saline intragastrically, while mice in experimental group were given corresponding concentration of saline intragastrically once a day for 14 days.

### 2.5 Observation

The dorsal skin of BALB/c mice was photographed every day, and the degree of skin lesions was scored. The severity of the skin lesions was observed by the naked eye, and the atopic/eczema dermatitis syndrome score was used to classify the skin lesions into four levels for dryness/desiccation, erythema/bleeding, and epidermal exfoliation/erosion: 0 (asymptomatic); 1 (mild); 2 (Medium); 3 (Heavy) [27], and the thickness of auricle was detected. The percutaneous dehydration of mouse dorsal skin was measured by a percutaneous dehydration instrument every 2 days.

### 2.6 Histological Analysis

On Day 15, the body weight, skin photo, and percutaneous dehydration test were recorded, and then the mice were killed. Take dorsal skin, local skin (about 2 × 2 cm^2^) in the same position, fix it in tissue fixative (4% paraformaldehyde), and store the rest skin at −80°C for later use. After routine dehydration, paraffin embedding, slicing and hematoxylin–eosin (HE) staining, the pathological changes of dorsal skin tissue were observed under optical microscope. The damaged skin tissue in the back of each group of mice was sliced, dewaxed, and aniline blue dyeing, washing, drying, transparent, and sealing. The infiltration of mast cells in dermal layer of mouse skin was observed under microscope, and the images were collected and analyzed.

### 2.7 Enzyme‐Linked Immunosorbent Assay

Mouse eyeball blood was taken. The blood was allowed to stand at room temperature for 2 h, and 1500 g was centrifuged at room temperature for 15 min. The serum was divided into two tubes and stored at −80°C. Serum immunoglobulin E levels and cytokine levels in skin tissue supernatant such as IL‐4, IL‐6, IL‐31, IFN‐γ, ET‐1, VEGFA, and TRPV1 were detected by enzyme‐linked immunosorbent assay.

### 2.8 16S rDNA Amplicon Sequencing

Microbial DNA is extracted from fecal samples using a fecal DNA kit. The V3‐V4 region of bacterial 16S ribosomal RNA gene was amplified by mobile primer PCR: 338F5 ′‐actCCtacGGgaggCAGCAGCAG‐3′, 806R5 ′‐GGACTACHVGGGTWTCTAAT‐3′. The PCR products were then extracted from 2% agarose gel for further purification and quantification.

The purified DNA amplification products were connected to the Illumina adapter (TruSeq DNA LT Sample Prep Kit) by ligating method, and the connected DNA fragments were subjected to equal molecular mixing and paired end sequencing (2 × 300) on the Illumina MiSeq platform. Sequencing was performed according to the standard process of Shanghai Mabo Bio‐Pharm Technology Co. LTD. According to the manufacturer's instructions (Illumina, San Diego, CA, USA), the DNA library was multiplexed and loaded onto the Illumina MiSeq instrument. Data analysis was conducted on the online platform of Majorbio Cloud platform (www.majorbio.com). In order to obtain the species classification information corresponding to each OTU, the RDP classifier was used to classify and analyze the representative sequences of each classification level (domain, boundary, phylum, class, order, family, genus, and species) at 97% similarity level, and the community species composition of each sample was counted. Mothur 1.30.2 software was used for Alpha diversity analysis and Qiime 1.9.1 software was used for beta diversity analysis. Finally, univariate analysis of variance was used to detect species with different abundances in each group.

### 2.9 Statistical Analysis

Generally, variance analysis is adopted, but it is necessary to test the homogeneity of variance according to the procedure of variance analysis, and calculate the *F* value, which is < 0.05—Conclusion: there is no significant difference between the mean values of each group; two independent samples *t*‐test (one‐way ANOVA, Bonferroni method, or LSD method) is used for groups of independent measurement data with *F* value ≥ 0.05 and *p* ≤ 0.05, which satisfy both normal distribution and homogeneity of variance. Nemenyi method was used for comparison between groups. Perform appropriate variable transformation on the data that is not normal or has uneven variance, and use the transformed data for statistics after meeting the requirements of normality or uniformity of variance; If the purpose of normality or homogeneity of variance is not achieved after the transformation of variables, the rank sum test is used for statistics.

## 3 Result

### 3.1 The Prescription Can Alleviate Skin Lesion Symptoms in AD Mouse Model

**FIGURE 1:**
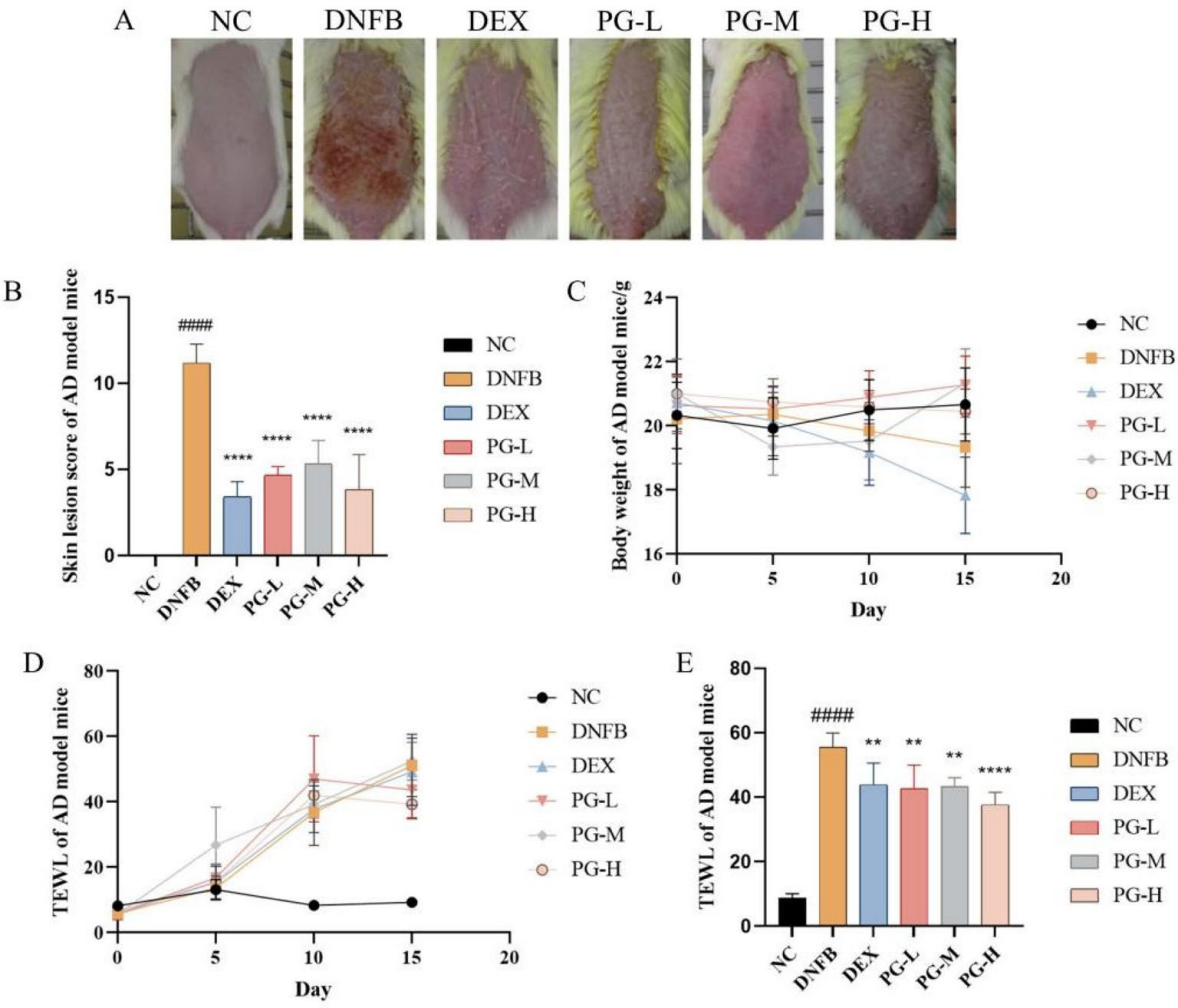
Effect of prescription on AD symptoms in DNFB induced mouse model. (A) Skin lesions on the back of mice before dissection; (B) Skin lesion score of mouse dorsal skin; (C) Weight of mice at Days 0, 5, 10, and 15; (D) TEWL of mice was measured on Days 0, 5, 10 and 15; (E) TEWL of dorsal skin of mice before dissection. All values are represented as means ± SEM (*n* = 3). ^####^ *p* < 0.0001, compared with NC group; ****p < 0.0001, **p < 0.01, compared with DNFB group.

After 14 days of modeling and drug administration, the DNFB group exhibited significant skin damage characterized by extensive papules, erythema, and scab‐like formations. In contrast, mice treated with various concentrations of the prescription group (PG) and the DEX group showed marked improvement in skin‐like symptoms (Figure 1A). The severity of skin lesions in each group was scored, with higher scores indicating more severe symptoms. The DNFB group's score was significantly higher than that of the NC group, while the scores of the treatment groups were notably lower than those of the DNFB group (Figure 1B). This demonstrates that oral administration of the formulation effectively alleviated skin redness, swelling, and scabbing.

### 3.2 The Prescription Can Reduce TEWL in AD Mouse Model

During the experimental period, the body weight of the mice was monitored (Figure 1C). The weight of the mice in each group showed little change throughout the experiment, although a downward trend was observed in the model group and the positive control group. As shown in Figure 1D, during the modeling process, TEWL levels were significantly elevated in all groups except the blank control group. By Day 15, a significant difference in TEWL was observed between the NC group and the DNFB group. Both the DEX group and the sample groups effectively inhibited the increase in TEWL, with the high‐concentration formulation demonstrating particularly notable efficacy.

The ability of the PG groups, especially the high‐concentration prescription, to suppress TEWL elevation highlights their potential to restore and enhance the skin barrier function. By improving skin hydration and reducing water loss, these formulations offer promising avenues for alleviating the discomfort associated with sensitive skin and improving overall skin health [28].

### 3.3 The Formulation Reduces Dorsal Skin Thickness, Ear Swelling, and Mast Cell Infiltration in AD Mouse Model

**FIGURE 2:**
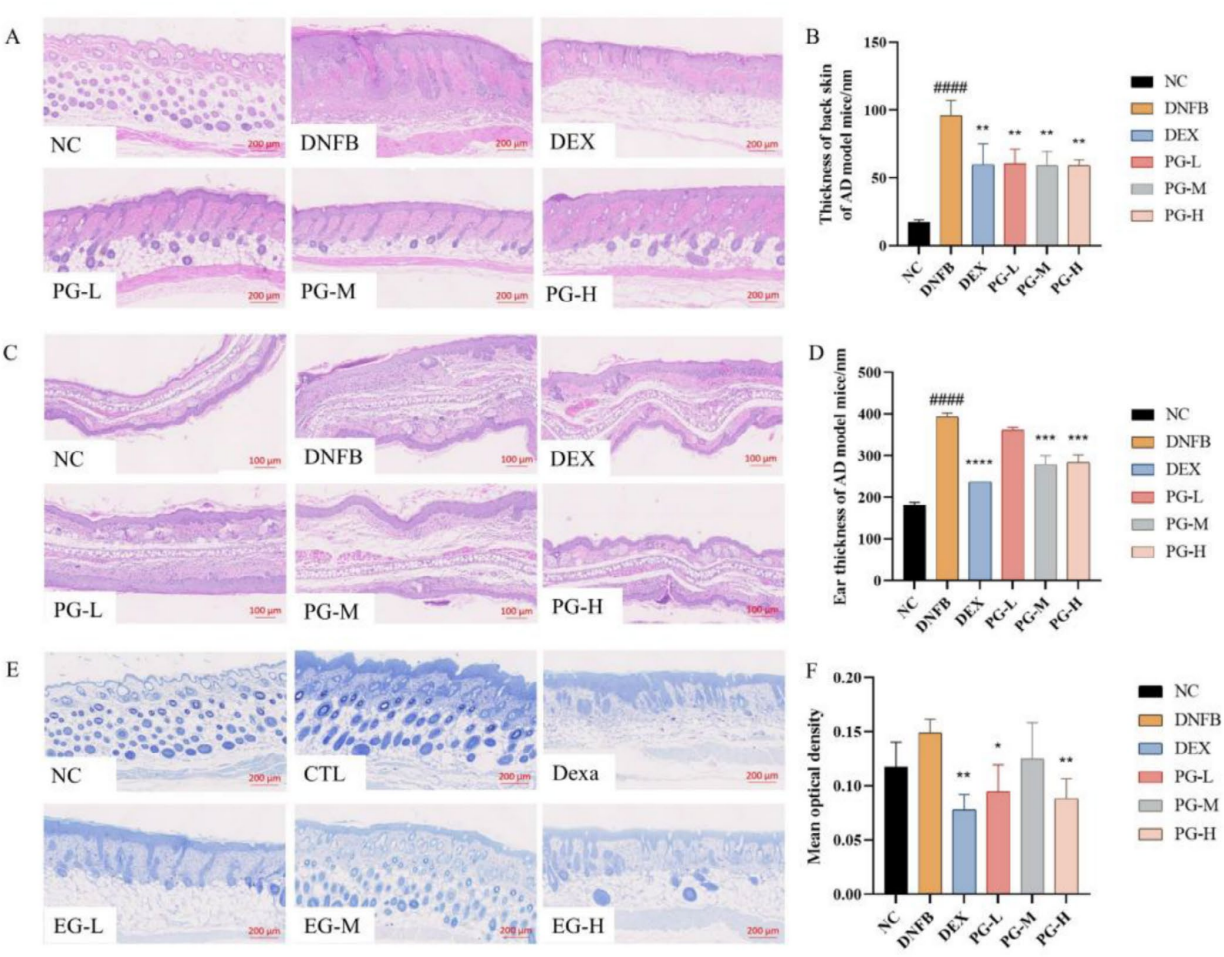
Effect of prescription on dorsal skin and ear tissue histopathology of AD mice. (A) HE staining of dorsal skin tissues (scale bar, 200 μm); (B) Epidermal thickness was measured using ImageJ software; (C) HE stained section of ear tissue (scale bar, 100 μm); (D) Ear skin epidermal thickness was measured using ImageJ software; (E) Staining of mast cells in the dorsal skin tissue (scale bar, 200 μm); (F) Calculate the number of mast cells at random locations of skin tissue. All values are represented as means ± SEM (*n* = 3). ^####^ *p* < 0.0001, compared with normal control group; *p < 0.05, **p < 0.01, ***p < 0.001, ****p < 0.0001, compared with DNFB group.

In this study, DNFB modeling induced abnormal epidermal hyperplasia and significantly increased skin thickness (Figure 2A), mimicking the hyperkeratosis of the skin observed in sensitive skin [29]. Oral administration of the positive control drug and the test prescription effectively inhibited the increase in epidermal thickness (Figure 2B), suggesting their potential to repair the skin barrier. HE staining was used to evaluate changes in auricle thickness and swelling, which reflect the intensity of the inflammatory response and skin barrier function [30]. After DNFB modeling, the auricle exhibited significant swelling and thickening (Figure 2C), with a statistically significant difference between the NC group and the DNFB group. Except for the PG‐M group, all other PG groups demonstrated notable efficacy in alleviating auricle swelling (Figure 2D). Toluidine blue staining was employed to assess mast cell infiltration [30]. DNFB modeling significantly increased the number of mast cells in the lesional skin, while the DEX group effectively reduced this infiltration. Among the PG groups, both the PG‐H and PG‐L groups exhibited significant suppression of mast cell infiltration (Figure 2E,F). These results indicate that the test formulation, particularly at higher concentrations, can mitigate key features of sensitive skin, such as epidermal hyperplasia, inflammation, and mast cell infiltration.

### 3.4 The Prescription Can Reduce the Levels of Inflammatory Cytokines in the Dorsal Skin of AD Mouse Model

**FIGURE 3:**
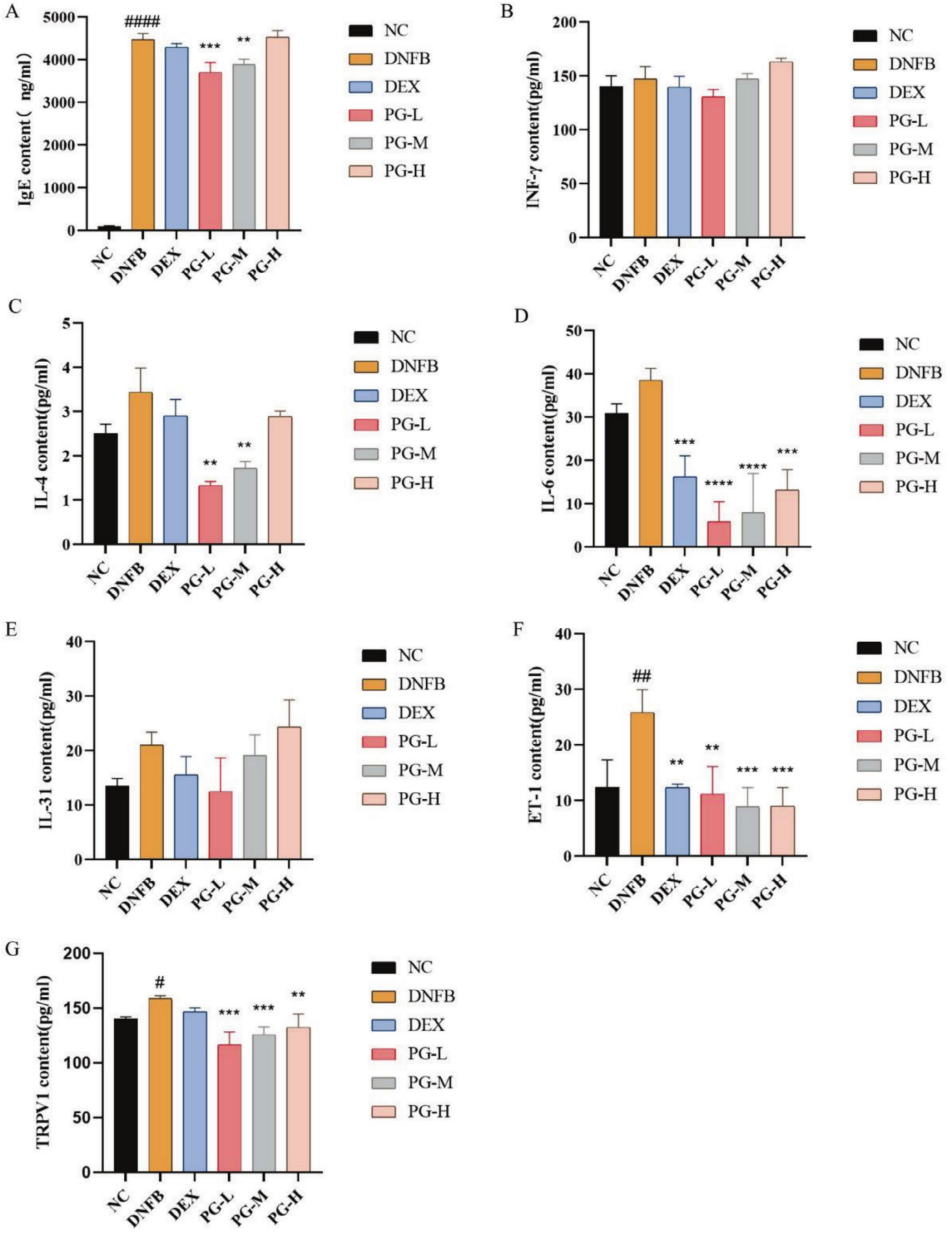
Effect of prescription on the levels of immunoglobulins in serum and cytokines in dorsal skin. (A) Levels of IgE in serum; (B) Levels of IFN‐γ in dorsal skin; (C) Levels of IL‐4 in dorsal skin; (D) Levels of IL‐6 in dorsal skin; (E) Levels of IL‐31 in dorsal skin; (F) Levels of ET‐1 in dorsal skin; (G) Levels of TRPV1 in dorsal skin. All values are represented as means ± SEM (*n* = 3). ^#^ *p* < 0.05, ^##^ *p* < 0.01, ^####^ *p* < 0.0001, compared with normal control group; **p < 0.01, ***p < 0.001, ****p < 0.0001, compared with DNFB group.

After modeling with DNFB, the expression levels of factors related to inflammatory responses, neural and vascular responses, such as IL‐4, IL‐6, IL‐31, IFN‐γ, IgE, TRPV1, and ET‐1, all increased. Following treatment with the prescription, the level of IL‐6 was significantly suppressed, while the low and medium concentrations of the formulation markedly reduced the levels of IL‐4 and IgE. It is hypothesized that the prescription may regulate the secretion of Th2/Th1 cytokines and modulate immune responses (Figure 3A,C,D). Concurrently, the treatment with the prescription also resulted in decreased levels of TRPV1 and ET‐1, indicating that the prescription can alleviate itching and reduce redness and swelling by modulating neurovascular responses (Figure 3F,G). These findings suggest that DNFB treatment may exacerbate inflammatory responses and barrier function damage in sensitive skin, whereas dexamethasone and the formulated prescription (particularly at medium and low concentrations) may help mitigate inflammatory responses and enhance skin barrier function.

### 3.5 The Prescription Modulates the Composition and Abundance of Gut Microbiota in AD Mouse Model

**FIGURE 4:**
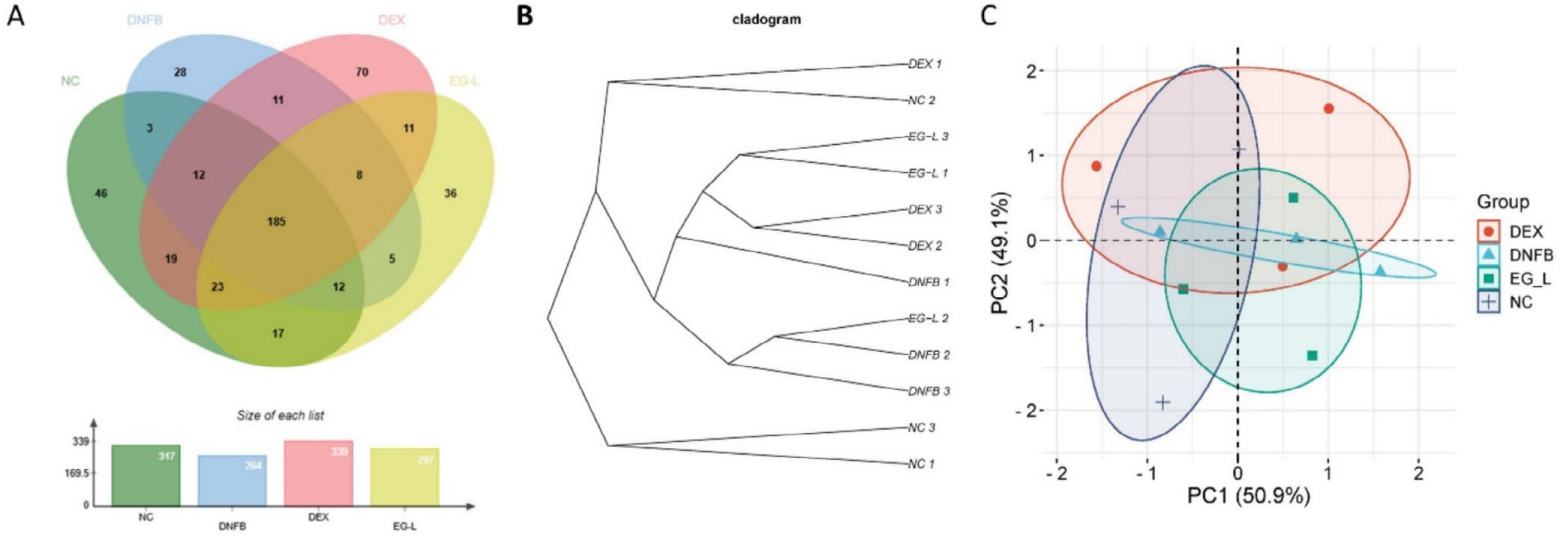
The influence of prescription on the taxonomic composition of the gut microbiota. (A) OTU Venn diagram with relative abundance > 0.1%; (B) UPGMA clustering results; (C) PCA plot with unweighted UniFrac.

To study the effects of the formulation on the entire gut bacterial community, we sequenced the 16S RNA in the feces of mice from the NC, DNFB, DEX, and EG‐L groups [31]. A Venn diagram shows the unique and common OTUs among the groups, with a relative abundance of > 0.1% (Figure 4A). As shown, the majority of OTUs (185 in total) were present in all four groups, with the unique OTUs for the NC, DNFB, DEX, and EG‐L groups being 46, 28, 70, and 36, respectively. Next, we assessed changes in gut microbiota diversity among the different groups. First, principal coordinate analysis based on an unweighted UniFrac distance matrix was performed (Figure 4C). Then, UPGMA clustering analysis using the unweighted UniFrac method was conducted, and the results are displayed as a dendrogram (Figure 4B). Subsequently, the Chao1 index, Simpson index, and Shannon index were used to evaluate the alpha diversity of the gut ecosystem (Table 2). Higher Chao1, Simpson, and Shannon values indicate a greater total number of species and higher community diversity [32]. In summary, the bacterial composition of the DEX, DNFB, and EG‐L groups showed separation, and there were differences in bacterial communities among the groups. This prescription can improve the composition of gut microbiota in mice, and these changes are closely related to skin health, potentially reducing skin inflammation and enhancing barrier function through the modulation of the gut‐skin axis, thereby exerting positive effects on skin diseases.

**TABLE 2:** Effects of prescription on the α‐diversity index of gut microbiota.

	PD_whole_tree	Chao1	Dominance	Observed_species	Shannon	Simpson
NC	6 ± 1	214.33 ± 22.18	0.13 ± 0.04	197.33 ± 30.75	4.37 ± 0.36	0.87 ± 0.04
DNFB	4.67 ± 0.58	169.4 ± 10.3	0.25 ± 0.13	147 ± 12	3.27 ± 0.78	0.75 ± 0.13
DEX	6 ± 0	210.88 ± 22.38	0.13 ± 0.03	181.67 ± 19.09	3.92 ± 0.48	0.87 ± 0.03
EG‐L	5.33 ± 0.58	199.28 ± 24.53	0.21 ± 0.04	177.67 ± 22.9	3.51 ± 0.51	0.79 ± 0.04

**FIGURE 5:**
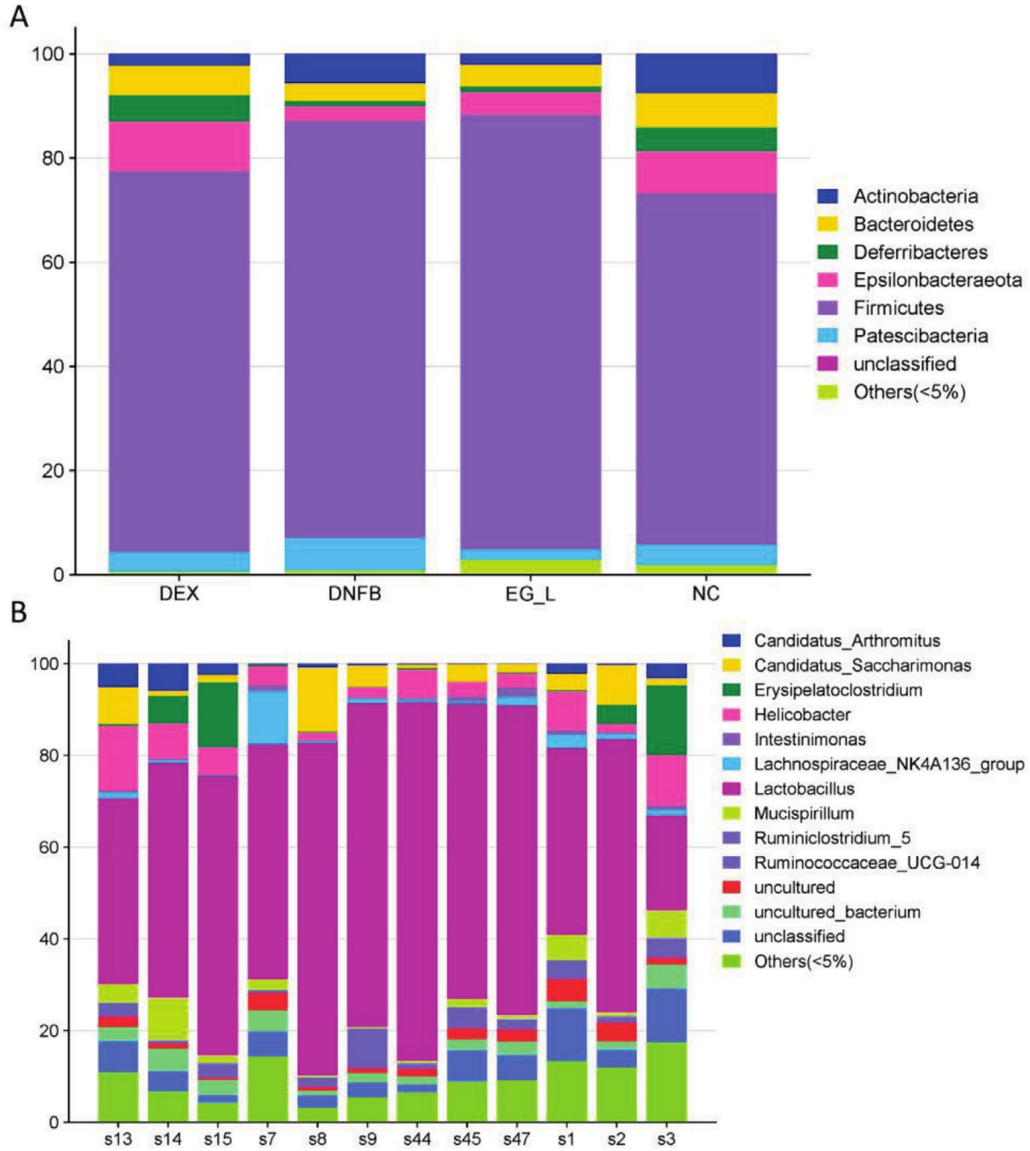
Influence of relative abundance of gut microbiota at (A) phylum level; (B) genus level.

Based on taxonomic analysis, the relative abundances of various phyla were calculated. We found that Firmicutes constituted the largest proportion of the gut microbiota, followed by Epsilonbacteraeota and Bacteroidetes (Figure 5A). The F/B ratio (Firmicutes to Bacteroidetes ratio) in the gut microbiota of the DNFB group was higher than that in the NC group. The treatment with the prescription stimulated the expansion of the Bacteroidetes population, leading to a decrease in the F/B ratio. Patients with sensitive skin often exhibit an imbalance in the Firmicutes/Bacteroidetes ratio, which may contribute to inflammation and impaired barrier function [32]. Taxonomic analysis also revealed that after 2 weeks of oral administration of the prescription, it suppressed the DNFB‐induced increase in Candidatus_Saccharimonas, Lachnospiraceae_NK4A136_group, and Ruminococcaceae_UCG‐014, as well as the decrease in Helicobacter and Mucispirillum (Figure 5B). These data indicate that the prescription treatment significantly modulates the composition of the gut microbiota in mice, playing an important role in treating skin barrier function and regulating immune responses. In summary, the prescription, by regulating the composition of the gut microbiota—particularly the Firmicutes/Bacteroidetes ratio—shows potential therapeutic effects in improving skin barrier function and immune regulation.

### 3.6 The Prescription Alleviates Dry Skin Symptoms and Reduces TEWL in AEW Mouse Model

**FIGURE 6:**
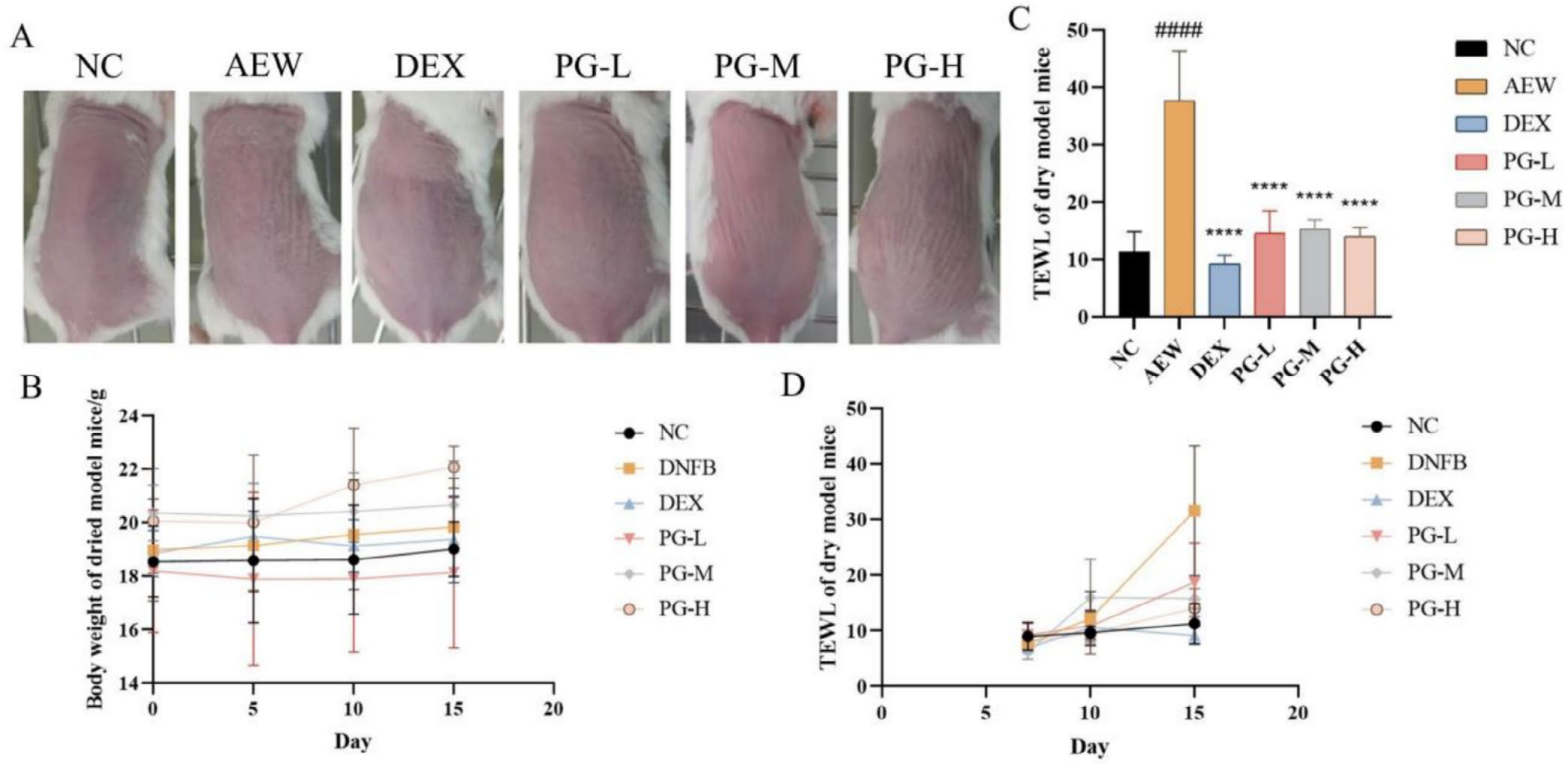
Effect of prescription on symptoms and skin physiological parameters in AEW mice. (A) Skin lesions on the back of AEW mice before dissection; (B) Weight of mice at Days 0, 5, 10, and 15; (C) TEWL of dorsal skin of mice before dissection; (D) TEWL of mice was measured on Days 8, 11 and 14. All values are represented as means ± SEM (*n* = 3). ^####^ *p* < 0.0001, compared with NC group; ****p < 0.0001, compared with AEW group.

After modeling, the skin on the backs of the mice became dry and developed some scales, particularly in the AEW group, where the scaling was more pronounced compared to other groups (Figure 6A). Body weight measurements taken on days 0, 5, 10, and 15 showed minimal changes in most groups, except for the high‐dose experimental group (Figure 4B). Notably, TEWL increased significantly during the modeling process, but the application of different concentrations of the test samples significantly inhibited the rate of TEWL (Figure 6D). On the 15th day, TEWL was measured in each group, and the treatment groups significantly suppressed the increase in TEWL (Figure 6C). These results are highly relevant to sensitive skin, as the observed dryness, scaling, and elevated TEWL reflect the impaired barrier function and heightened reactivity characteristic of sensitive skin [28]. The ability of the test samples to reduce TEWL suggests their potential to restore skin barrier integrity and alleviate symptoms associated with skin sensitivity.

### 3.7 The Prescription Reduces Skin Thickness in AEW (Acetone‐Ether‐Water) Mouse Model

**FIGURE 7:**
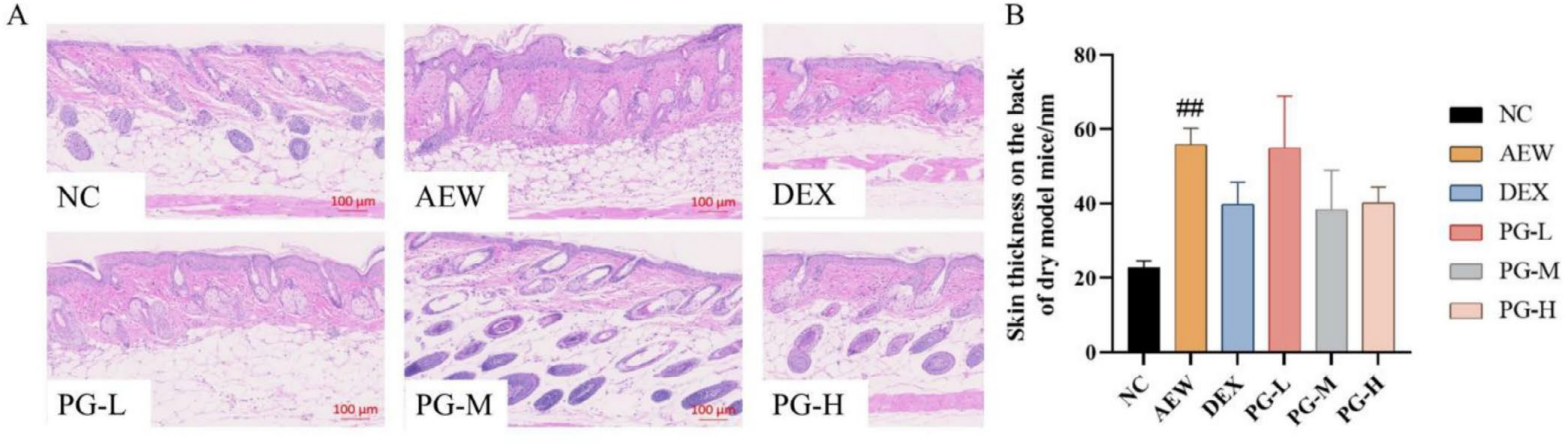
Effect of prescription on skin tissue histopathology of AEW mice. (A) HE staining of dorsal skin tissues (scale bar, 100 μm); (B) Epidermal thickness was measured using ImageJ software. All values are represented as means ± SEM (*n* = 3). ^##^ *p* < 0.01, compared with normal control group.

The morphological changes in the epidermis were observed using hematoxylin and eosin (HE) staining. Following the modeling process, the epidermis exhibited abnormal proliferation, accompanied by a significant increase in thickness, which markedly differed from the normal skin structure. Although the prescription demonstrated inhibitory effects on the abnormal thickening of the epidermis, these effects were not statistically significant (Figure 7A,B). These findings are closely related to sensitive skin, as abnormal epidermal proliferation and increased skin thickness are common features of skin barrier dysfunction, often observed in sensitive skin conditions [33]. The tendency of the prescription to reduce epidermal thickening suggests its potential to modulate skin barrier repair and alleviate the structural abnormalities associated with sensitive skin.

### 3.8 The Prescription Can Enhance the Expression Levels of Proteins Closely Related to Skin Barrier Function in the AEW Mouse Model

**FIGURE 8:**
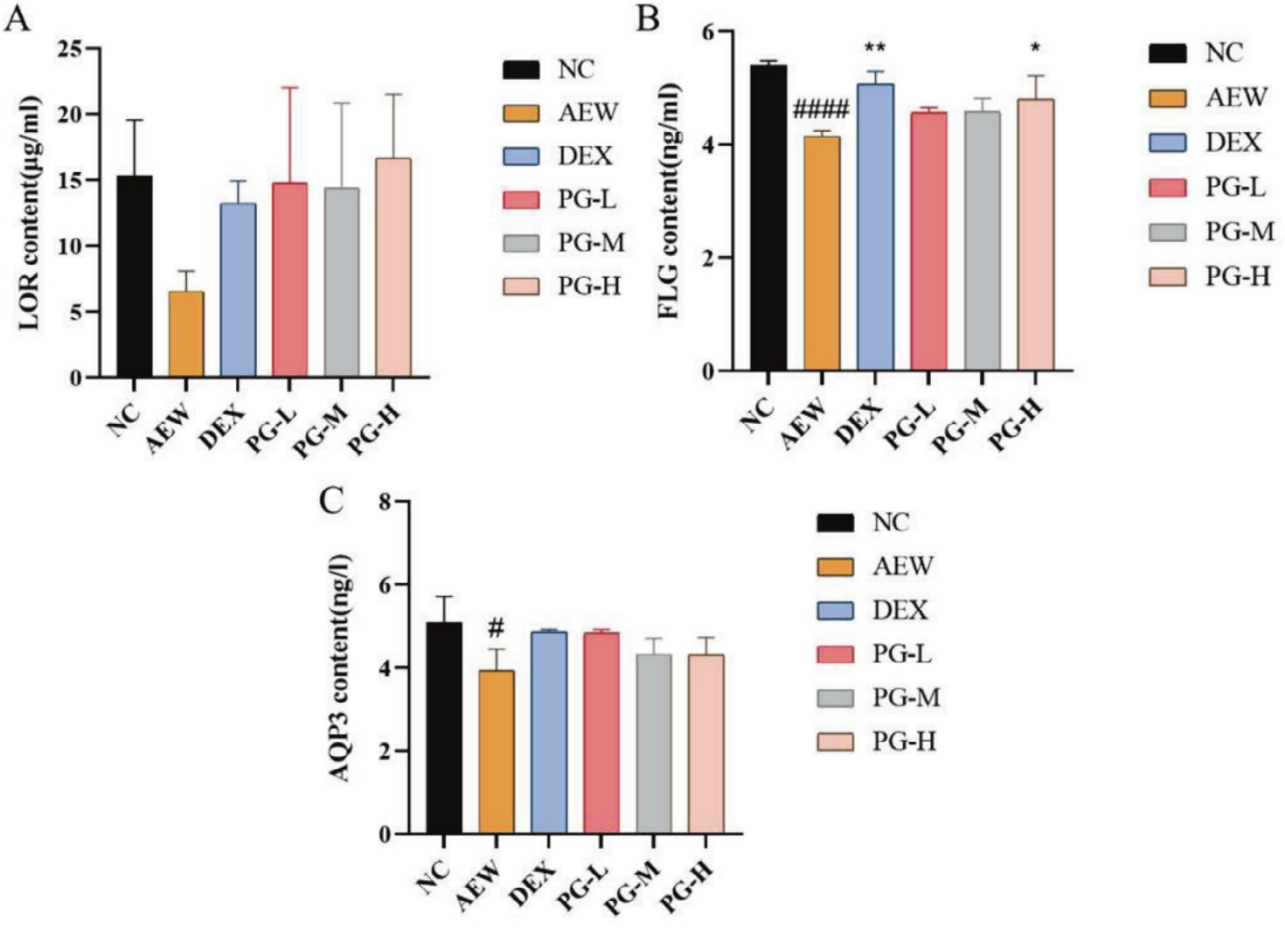
Effect of prescription on the levels of (A) LOR; (B) FLG; (C) AQP3 in dorsal skin. All values are represented as means ± SEM (*n* = 3). ^#^ *p* < 0.05, ^####^ *p* < 0.0001, compared with NC group; *p < 0.05, **p < 0.01, compared with AEW group.

Following treatment with acetone and ether, the levels of cytokines related to skin barrier function, such as FLG, AQP3, and LOR, were significantly decreased. However, after therapeutic intervention, the expression levels of LOR, filaggrin (FLG), and AQP3 were restored to varying degrees, indicating a repair and enhancement of the skin barrier (Figure 8A–C). The downregulation of LOR, FLG, and AQP3 is often linked to impaired barrier function, increased transepidermal water loss (TEWL), and heightened skin sensitivity [34]. The ability of the prescription to upregulate these critical proteins suggests its potential to restore skin barrier integrity, improve hydration, and reduce reactivity in sensitive skin.

## 4 Discussion

This study demonstrates that an oral prescription comprising ceramide, proanthocyanidins, quercetin, and citrus flavonoids significantly alleviates symptoms of sensitive skin and atopic dermatitis (AD) in murine models. Our results indicate that this formulation not only enhances skin barrier function by reducing transepidermal water loss (TEWL) and decreasing skin thickness but also exerts anti‐inflammatory effects, mitigating key inflammatory cytokines associated with AD pathogenesis. Furthermore, our findings suggest that the oral prescription has the potential to modulate the gut microbiota, a factor increasingly recognized as influencing skin health.

In the DNFB‐induced AD model, we observed that treatment with the oral prescription significantly reduced the severity of skin lesions, as evidenced by a decrease in lesion scores, TEWL, and epidermal thickness. These results are consistent with previous studies demonstrating that ceramide‐based formulations improve skin barrier function and reduce symptoms in AD models [23, 35, 36]. The improvement in TEWL, a critical marker of skin barrier dysfunction, further supports the hypothesis that the oral prescription helps restore barrier integrity. The reduction in epidermal thickness and ear swelling in our study suggests that the prescription also alleviates the hyperproliferation of keratinocytes, a hallmark of AD skin lesions [37, 38, 39].

Interestingly, the anti‐inflammatory effects observed in this study appear to be mediated through the modulation of several key cytokines implicated in AD. Specifically, the reduction in IL‐4, IL‐6, IL‐31, and ET‐1 expression following treatment suggests a potential Th2 immunomodulatory effect of the formulation. IL‐4 and IL‐6 are involved in the pathogenesis of AD by promoting the production of IgE, increasing inflammation, and impairing the skin barrier. Additionally, IL‐31, a cytokine linked to pruritus and skin inflammation, was also significantly reduced, further highlighting the therapeutic potential of the formulation in managing itching and inflammation associated with AD. Although the formulation did not exhibit a strong inhibitory effect on IFN‐γ or IL‐31, its ability to suppress these pro‐inflammatory cytokines still indicates its overall anti‐inflammatory potential [40, 41, 42].

Our study also demonstrated that treatment with the oral prescription reduced mast cell infiltration in the skin, a hallmark of AD pathology. Mast cells release a variety of inflammatory mediators, such as histamine and cytokines, which exacerbate skin inflammation. The observed suppression of mast cell infiltration in the high‐concentration group (PG‐H) suggests that the formulation may help prevent or reduce the inflammatory cascade initiated by these cells [43].

In addition to its anti‐inflammatory effects, we discovered that the oral prescription boosted the expression of key skin barrier proteins, such as filaggrin (FLG), aquaporin‐3 (AQP3), and loricrin (LOR). These proteins are crucial for maintaining the integrity and hydration of the skin barrier. In cases of atopic dermatitis (AD) and sensitive skin, a reduction in FLG and LOR levels is frequently observed, which contributes to compromised barrier function and heightened susceptibility to irritation. The replenishment of these proteins following treatment with the oral prescription indicates its potential to repair and fortify the skin barrier, further substantiating its therapeutic use in managing sensitive skin conditions [44, 45, 46].

Additionally, the impact of oral administration on the composition of the gut microbiota was also assessed. The results indicated that the oral formulation altered the abundance of Firmicutes and Lactobacillus in the gut, which has previously been associated with skin health. Research has demonstrated that dysbiosis in the gut microbiome can affect the onset and progression of atopic dermatitis (AD) [10, 47, 48], and our findings suggest that the oral prescription might exert a systemic effect by modulating the gut‐skin axis. However, although the observed changes in microbiota composition were not statistically significant, they merit further investigation, especially in clinical trials, as the role of the microbiome in the treatment of sensitive skin and AD is an area of increasing interest.

In the AEW‐induced dry skin model, oral administration was similarly effective in reducing transepidermal water loss (TEWL) and enhancing skin hydration, suggesting that it can restore skin barrier integrity in a model of noninflammatory skin dryness. These findings are consistent with previous reports indicating that flavonoids, such as quercetin and citrus flavonoids, possess antioxidant and anti‐inflammatory properties that support barrier function and prevent dehydration [49].

Overall, the oral prescription comprising ceramide, proanthocyanidins, quercetin, and citrus flavonoids presents a promising therapeutic strategy for managing sensitive skin and atopic dermatitis (AD). Its multifaceted effects, which include skin barrier restoration, inflammation modulation, and potential gut microbiota modulation, make it a compelling option for treating these chronic and often recalcitrant conditions. Further clinical studies are necessary to confirm its efficacy in humans and to explore the mechanisms underlying its therapeutic effects.

## 5 Conclusion

In summary, this formulation contains ceramides, proanthocyanidins, quercetin, and citrus flavonoids, demonstrating significant therapeutic potential for sensitive skin. The formulation can inhibit the transcription of pro‐inflammatory cytokines (such as IL‐6 and IgE), effectively alleviating skin inflammation while restoring levels of skin barrier proteins to improve barrier function and enhance TEWL, further reducing skin inflammation. Additionally, the active ingredients in this formulation may strengthen their positive impact on skin health by modulating gut microbiota diversity. These findings suggest that the combination of these active ingredients represents a promising approach for developing topical treatments for sensitive skin. Further clinical studies are needed to confirm the efficacy and safety of this formulation in humans.

## Author Contributions

M.Y., Y.Y., and S.C. performed the research. M.Y., C.W., P.H., and L.J. designed the research study. C.W., G.Y., X.Y., and Z.D. contributed essential reagents or tools. C.W. and N.J. analyzed the data. M.Y., L.X., and J.C. wrote the paper.

## Ethics Statement

The animal study was reviewed and approved by the Laboratory Animal Ethics Committee (NO. IACUC‐AEWC‐F240604006) and took place in the SPF Animal Laboratory at Guangzhou Forevergen Biosciences.

## Conflicts of Interest

The authors declare no conflicts of interest.

## Data Availability

The data that support the findings of this study are available from the corresponding author upon reasonable request.
